# Covalent Organic Frameworks: New Materials Platform for Photocatalytic Degradation of Aqueous Pollutants

**DOI:** 10.3390/ma14195600

**Published:** 2021-09-27

**Authors:** Yuhang Qian, Dongge Ma

**Affiliations:** Department of Chemistry, College of Chemistry and Materials Engineering, Beijing Technology and Business University, Beijing 100048, China; 2030401014@st.btbu.edu.cn

**Keywords:** covalent organic frameworks (COFs), photocatalysis, photodegradation, dyes, antibiotics, pharmaceuticals

## Abstract

Covalent organic frameworks (COFs) are highly porous and crystalline polymeric materials, constructed by covalent bonds and extending in two or threedimensions. After the discovery of the first COF materials in 2005 by Yaghi et al., COFs have experienced exciting progress and exhibitedtheirpromising potential applications invarious fields, such as gas adsorption and separation, energy storage, optoelectronics, sensing and catalysis. Because of their tunablestructures, abundant, regular and customizable pores in addition to large specific surface area, COFs can harvest ultraviolet, visible and near-infrared photons, adsorb a large amount of substrates in internal structures and initiate surface redox reactions to act as effective organic photocatalysts for water splitting, CO_2_ reduction, organic transformations and pollutant degradation. In this review, we will discuss COF photocatalysts for the degradation of aqueous pollutants. The state-of-the-art paragon examples in this research area will be discussed according to the different structural type of COF photocatalysts. The degradation mechanism will be emphasized. Furthermore, the future development direction, challenges required to be overcome and the perspective in this field will be summarized in the conclusion.

## 1. Introduction

As human industrialization and civilization processes accelerate and advance rapidly, the excessive dependence on the utilization of fossil fuels has lead to an energy crisis and environmental pollution [1]. These two issues are the major threats tothe sustainable development of human society. Sunlight, as a green and inexhaustiblerenewable energy, is considered to be the most ideal choice to substitute fossil fuels with and counter theenergy crisis and environmental pollution. Photovoltaic, photothermaland photocatalysis are the three main means with which to exploit the energy of sunlight. Photocatalysis, a process regarded as“artificial photosynthesis”, can transform less utilizable sunlight energy into utilizable chemical energy by photocatalyticly splitting water into H_2_ and O_2_, reducing CO_2_ into valuable chemicals such as methane and methanol, converting less valuable raw chemicals into high-value-added products by catalytic photochemical reactions. Moreover, photocatalysis can also be applied in the degradation of variouspollutants. As a type of advanced oxidation technology, photocatalysts can produce highly oxidative species such as valence band holes and secondary reactive oxygen species (ROS) such as O_2_^−^^•^, ^•^OH, ^•^OOH and H_2_O_2_. These strongly oxidative species can be utilized to degrade organic dyes, pharmaceuticals, antibiotics, herbicides and agrochemicals in sewage by consecutive hydroxylation and cleavage of the pollutants’ aromatic cyclic structure and generating innocuous CO_2_, H_2_O and inorganic salt degradation products. On the other hand, the reductive conduction-band electrons can also be applied to reduce toxic high-valenceheavy metal ions to less toxic low-valence ions such as Cr^6+^ to Cr^3+^. Fujishima and Honda et al. first discovered the photocatalysis phenomena that TiO_2_ can be incorporated into a photoelectrochemical device to evolve H_2_ and O_2_ under UV light irradiation [2]. Only several years later, Carey et al. discovered that TiO_2_ and ZnO photocatalysts can be used to degrade and mineralize pollutants under lightirradiation [3]. These pioneering works stimulated continuous enthusiasm to develop more effective photocatalysts for the purpose of watersplitting, CO_2_ reduction and utilization, organic transformation and pollutant degradation. Among the various photocatalysts, metaloxides (TiO_2_, ZnO, Fe_2_O_3_, BiOX and BiVO_4_) [4,5,6,7] and metalsulfides (CdS, MoS_2_ and ZnInS_2_) [8,9] are the most widely used and investigated photocatalysts due to their satisfactory photocatalytic activity under UV or visible-light irradiation, simple composition and convenient preparation procedures. However, these inorganic semiconductor photocatalysts usually suffer from an inefficient light-harvesting range (commonly less than 500 nm), severe electron-hole recombination, less surface area and unsatisfactory substrate adsorption properties.

Covalent organic frameworks (COFs) are a type of purely organic crystallineporous polymeric material [10]. Due to their high structural tunability, various functional building blocks can be incorporated into COFs to render theirdistinctphotoactivity [11]. The elaborate choice of functional blocks with various electronic and steric configurations can enlarge their absorption range and cover the sunlight spectrum from UV light to visiblelight to the infrared region. Furthermore, via bottom-up synthesis, post-modification, doping, photo-sensitization and hybridization with other photoactive compounds, the construction of the heterojunction structure, the electron-hole recombination can be greatly retarded, the charge transfer processes can be accelerated and the charge transfer resistance can be greatly decreased [12]. Moreover, the considerably large specific surface area and numerous regular and customizable micro- and mesopores can offer favorable guest-host interaction between substrates and catalytic sites. In order to build more suitable space for the guest-host interaction, the building blocks to produce such an organic porous architecture are generally rigid anddo not possess bulky side groups. The former is important to prevent the collapse ofthe structure through the empty voids (pores), and the latter is necessary to avoid poreblockage. Moreover, the polymerization direction is not typically linear; therefore, at leastone of the (co)monomers should be angled [13]. These abundant, porous and highly ordered structures can enhance the mass transport process inside the catalysts. The reactants and intermediates can be preferentially trapped and adsorbed into the internal pores and the products can be desorbed easily from catalysts to the bulk solution. The selectivity in the adsorption and desorption processes can be fine-tuned by the tactic design of the monomer building units, the linking reticular chemistry and the dynamic chemical reactions fabricating specific microenvironments with customizable size, polarity, hydrophobicity and electrostatic interaction [14]. With these advantages, COFs have promisingly been exhibited as ideal photocatalysts and garnered considerable success in various fields, including photocatalytic watersplitting [15], CO_2_ reduction [16], organic transformations [17] and pollutant degradation [18]. Especially for the degradation of pollutants in water, COF-based photocatalysts, despite a late start, show inherent advantages over traditional ones based on both inorganic and organic semiconductor photocatalysts. Besides well-knownhuge specific surface areas (caneasily reach 10^2^~10^3^ m^2^/g) and tailorable shape selectivity that can be favorable toaccumulate and degrade the targeted pollutants, with very trace distribution but very high toxicity from common water, COFs have the most advantage overother current photocatalysts towards mitigation of aqueous pollutants for their capability to minimize the competition of solvent H_2_O and other general dissolved organic matter (DOM) to active sites and reactivespecies via highly orderedπ-arrays and covalently fashionedpore structures. In contrast, neither inorganic star photocatalyst TiO_2_ normodified organicg-C_3_N_4_ photocatalysts directly react with trace organic pollutant substrates in water, instead, they mainly do so by means of subsequent species of H_2_O oxidation or O_2_ reduction, in order to diffuse into bulk solutions for the degradation of targetedpollutants [19,20,21], commonly lowering photocatalytic efficiency under otherwise identical conditions.

Although there are a number of excellent reviews focusing on photocatalysis by COF materials in panoramic views, considering the structure design, modification methods and various photocatalysis applications [19,20,22,23], there are still a lack of review articles specifically concentrating on the photocatalytic degradation of aqueous pollutants by COFs. Given the importance of this field, this review will provide the state-of-the-art examplesin this research area. The discussion will be categorized according to the different COF structuresand the degradation mechanisms will be emphasized. The challenges in this field in addition to the future development outlook and perspective will be offered in the conclusion part.

## 2. COFs Photocatalytic Applicationsin Degradation of Pollutants

### 2.1. The General Principle of Photocatalytic Active COFs and Their Stability in Water and Harsh Conditions

**Figure 1:**
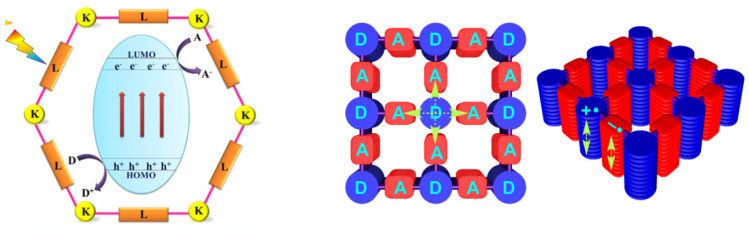
Schematic illustration of COF-based photocatalysts in which K and L represent knots and linkers, respectively (**left**) [12]; D and A represent donors and acceptors of COFs, respectively (**right**) [19]. (Copied with permission from RSC and ACS).

Usually, photocatalyticactive COFs consist of different functional molecular blocks (e.g., triazine-based, pyrene-based, porphyrin-based and thiophene-based COFs) as electron donors or acceptors, respectively, viathe linkage of covalent bondssuch asimine, imide, hydrazone, azine, boronate ester, alkene, etc. (Figure 1 [24]). According to band theory, the *Eg* of COF relies on the energy gap between its highest occupied molecular orbital (HOMO) and lowest unoccupied molecular orbital (LUMO) levels, which are the same meanings as VB and CB in inorganic semiconductors, respectively. The absorption of light takes place by the transition of electrons from the HOMO to the LUMO. Subsequent chargeseparationgenerates redox potential on VB-like and CB-like sites. This drives substrate molecules to perform chemical bond cleaving and form reactions in COFs surface and channels. Although the basic principle is very similar to that of conventional photocatalytic materials, the polygon channel structure and the periodically arranged pore walls provide COFswith well-defined nanospace as reaction centers, which enable fast immigration of excitons and chargeseparation. Therefore, COF materials as photocatalysts possess more advantagesover traditional photocatalytic materials. First, the topologies, channels and band structure of COFs can be easily tuned by introducing various molecular building blocks. Their permanent and nanoscale pore structure leads to large surface areas and thereby creates more active sites, permitting substrates facile access to photocatalytic active sites of COFs. Second, the COFs used as photocatalystsconsist of electron donor-acceptor (D-A) components (Figure 1). The transfer of electrons from donors to acceptors improves the separation efficiency of photo-generated electron-hole pairs. Third, the periodically ordered columnar arrays of the π-conjugated system are in favor of electronic delocalization and thus render COFs an excellent electrontransporting property and prominent photoconductivity [25]. Therefore, as soon as it was born, COF-based photocatalysis has inspired a new wave of research.

Not all COF materials with high photocatalytic activity, however, can be used in oxidative or reductive removal of pollutants in water. The stability of COFs or their usabilityin water and harsh conditions, such as solar irradiation, for crystalline materialsis more critical and even more fatal than photocatalytic efficiency. For example, despite the fact that boronate- andboroxine-linked COFs exhibited excellent photoconductive properties, they were seldom used to perform photocatalytic reactions in solutions because these types of linkage displayed poor stability under protic conditions or upon exposureto air [26]. Namely, many COFs show good thermostability, but their stability in aqueous solutions remainsa challenge. This stems from the inborn reversibility of most synthesis COF materials’ reactions (i.e., most COFs’hydrothermal synthesis involved dehydration condensation). Therefore, COFs will tend to decompose in an aqueous system. Compared to boroxine and boronate ester linkages, imine-linked COFs provide the network with rich electrons such that the structural resistance of COFs to water can significantly increase [27]. There have been some strategies to improve the stability of COFs in water and harsh conditions, such as acidic or alkaline solutions or a high-redox press environment. The measures include the following: (i) the introduction of intramolecular hydrogen bonds such as O–H⋯N=Cin the linkage structuremoiety of COFs to protect the basic imine nitrogen from hydrolysis in the presence of water and acid [28]; (ii) linkage conversion, such as transforming imine linkages of COFs into amide linkages by direct oxidationto improve water stability [29], or converting the enol-imine (OH) form into the ketonenamine form bythe irreversible proton tautomerism to endow COFs with strong resistance toward acid and even boiling water [30]; and (iii) combining COFs with other water-stable materials such as graphene oxide (GO) to fabricate hybrids in order to achievegood stability in water [31].

Now, according to the linkage categories with certain stabilities in water above-mentioned, we focus on the application of imine-based, ketonenamine-based and other stable-linkages-based COF photocatalysts as well as their composites with other semiconductors, such as inorganicsemiconductors, MOFs, g-C_3_N_4_, etc. in the degradation of pollutants in water.

### 2.2. Imine-Based COF Photocatalysis for Pollutant Degradation

Since the milestone discovery of COF-1 and COF-5 by Omar Yaghi et al. in 2005 [32], COF materials have undergone rapid and tremendous development [33]. In 2009, Yaghi et al. exploited the reversible Schiffbase condensation between aldehyde and primary amine to yield the C=N imine-linked COF-300 [34]. Unlike the boron-containing COFs, imine linkage exhibited much enhanced stability towards water and common organic solvents. In this way, Wang et al. first designed and prepared a 2D imine-linking COF-LZU-1 by the Schiffbase condensation of 1,3,5-benzenetrialdehyde with 1,4-diaminobenzene. The as-formed 2D COF-LZU-1 possessed a large surface area and was loaded with Pd nanoparticles conveniently. The Pd/COF-LZU-1 displays superior performance in catalyzing the Suzuki-Miyaura reaction between halobenzene with phenyl boronic acid comparedto the homogeneous organometallic Pd and the heterogeneous Pd/C catalysts [35]. The COF catalyst demonstrated its advantages in easy separation, recovery of catalyst and satisfactory reuse activity [19,24]. Furthermore, the pore structure is more beneficial for the approaching and reacting of organic substrates with catalytically active Pd species due to the hydrophobic microenvironment effects. Later on, by incorporating a photo-responsive buildingblock into the imineCOF backbone, photoactive catalytic functional COFs can be fabricated [36]. As pioneered COFs for catalytic applications, imineCOFs can harvest sunlight, generate electron and hole charge carriers, adsorb the reactants and lower the barrier and activation energy to initiate the redox reaction [37].

**Figure 2:**
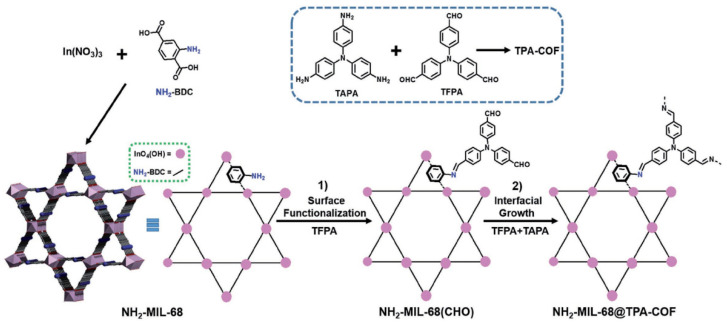
Schematic illustration of the synthesis of NH_2_-MIL-68@TPA-COF hybrid materials (copied with the permission of Wiley 2018 [38]).

In 2017, Zhang et al. utilizedimine-based COFs for the application of photocatalytic degradation of rhodamine B (RhB) organic dyes in water [38]. The authors discovered that, by the hybridization of an indium-based metal-organic framework (MOF) NH_2_-MIL-68 with an imine-based TPA-COF by a covalent bond linkage strategy, the core/shell MOF@COF composite photocatalyst can realize boosted performance for RhB photodegradation due to enhanced electron-hole separation by the as-formed heterojunction structure (Figure 2). This MOF@COF hybrid material exhibited higher degradation efficiency for RhB andreusabilitycompared with other MOF photocatalysts.

**Figure 3:**
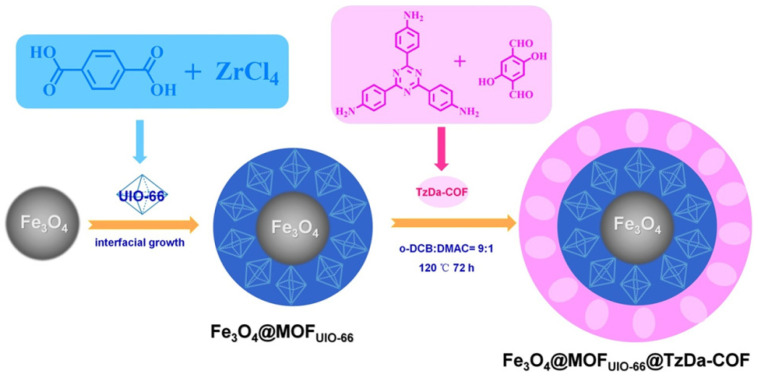
Schematic illustration of the preparation of Fe_3_O_4_@MOF_UiO-66_@Tz-Dz-COF matryoshka hybrid magnetic photocatalyst (copied with permission from ACS 2020 [39]).

Furthermore, Xu et al. reported that Fe_3_O_4_ supermagnetic nanoparticles, Zr-based UiO-66 MOF and imine-based TzDa-COF, can be merged into a Fe_3_O_4_@MOF_UiO-66_@TzDa-COF matryoshka structure (Figure 3) [39]. The as-synthesized matryoshka photocatalyst exhibited satisfactory rapid degradation of malachite green (MG) and Congo red (CR) anionic dye pollutants in an aqueous solution. The large surface area of MOF/UiO-66 and TzDa-COF contributed to the considerably fast adsorption and photodegradation. Moreover, the magnetic Fe_3_O_4_ core offered the convenient recycle and separation behaviors of catalysts from bulk solution. This matryoshka photocatalyst displayed superior activity upon MG and CR degradation in comparison to previously reported materials. This report was the first to hybridize MOF and COF photocatalysts with magnetic nanoparticles to fulfill better recycle and reuse performance.

**Figure 4:**
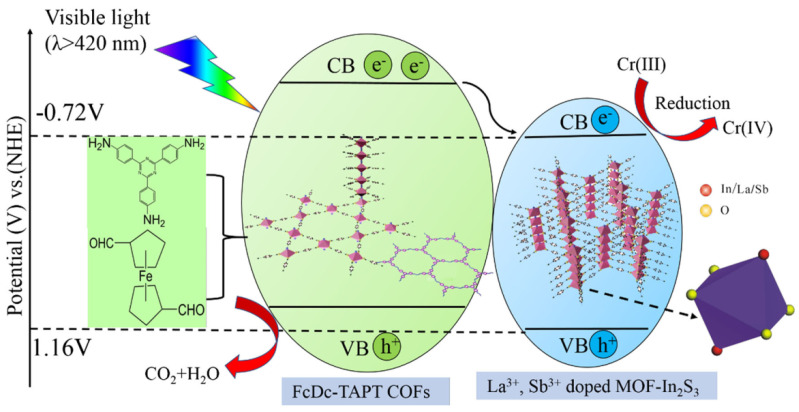
Schematic illustration of photocatalytic reduction of Cr (VI) over La^3+^, Sb^3+^-doped MOF-In_2_S_3_@FcDc-TAPT COFs under visible-light irradiation (copied with permission from Springer 2019 [40]).

Apart from MOF@COF composite photocatalysts, pure inorganic metal sulfide photocatalysts can also be incorporated into COF backbones to construct more effective photocatalysts for aqueous pollutant degradation. Pi et al. reported that by a stepwise strategy, La^3+^, Sb^3+^-NH_2_-MIL-68(In)-FcDc-TAPT-COF composite photocatalysts were fabricated [40]. This photoactive material was exploited for the reduction of toxic heavy metal Cr (VI) to less toxic Cr (III) under visible irradiation (Figure 4). The metal-ion-doped MOF@COF hybrid materials provided better performance in comparison to non-doped MOF@COF and separate MOF or COF materials. The electron impedance spectrometry and photoluminescence studies demonstrated that the MOF@COF structure could help to form the heterojunction to facilitate charge carrier transfer. Moreover, the loading of metal ions assisted to generate an electron trap in the band gap and inhibited the electron-hole recombination, thus increasing the electron density for more effective ROS generation and Cr (VI) photo-reduction.

**Figure 5:**
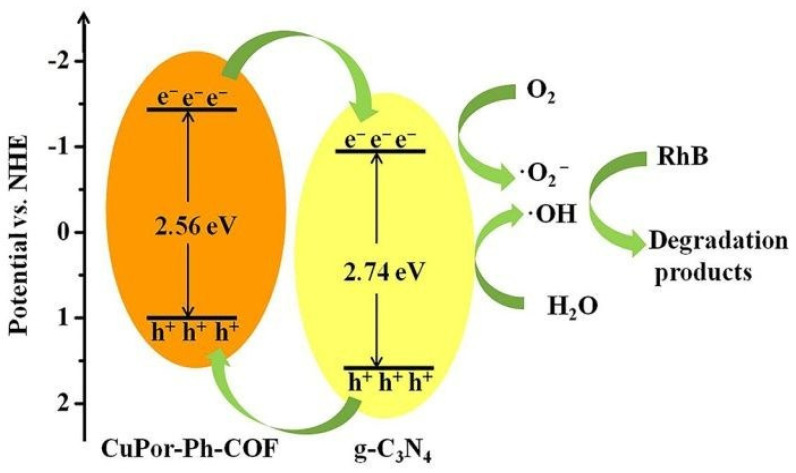
Schematic representation of the photodegradation of RhB by CuPor-Ph-COF@g-C_3_N_4_ composite photocatalyst. (Copied with permission from RSC 2019 [41]).

Besides inorganic and inorganic-organic hybrid photoactive materials, imine COFs can also merge with pure organic semiconductor photocatalysts, such as graphitic carbon nitride (g-C_3_N_4_), to yield heterojunctions and provide superior photoactivity. Wang et al. reported that by a convenient liquid-assisted grinding procedure, CuPor-Ph-COF can be prepared on the surface of g-C_3_N_4_ to form a heterojunction photocatalyst [41]. The composite photocatalyst exhibited superior activity towards the degradation of RhB dye pollutant compared to the pristine g-C_3_N_4_ and CuPor-Ph-COF materials. Moreover, as evidenced by the radical scavenging experiments, the main species that accounted for the degradation were determined to be ^•^OH, superoxide and e^−^ (Figure 5). This report was the first to demonstrate the capability of forming porphyrin-based imine COFs with g-C_3_N_4_ materials for more efficient organic dye elimination via enhanced visible-light absorption and more effective electron-hole separation and transfer. In addition, incorporation of g-C_3_N_4_ into the composite photocatalyst remarkably improved the stability of imine-based COFs in water solutions.

**Figure 6:**
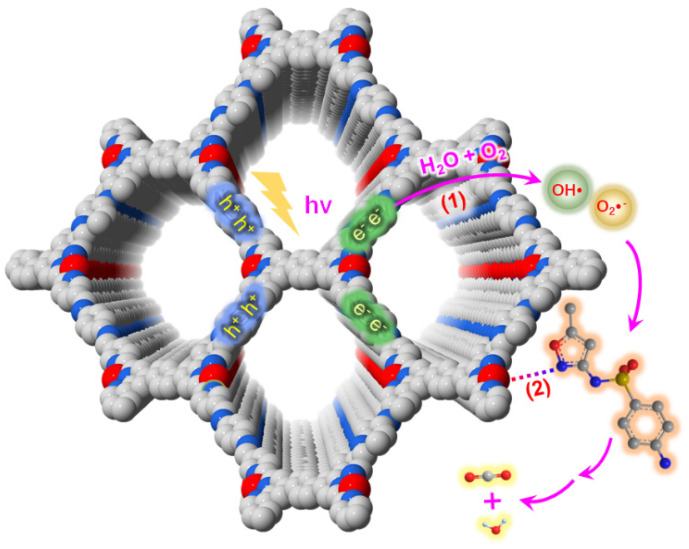
Schematic illustration of the mechanism of photocatalytic degradation of SMX using COF-909 (Cu). (Copied with permission from Elsevier 2021 [42]).

Not only can inorganic and organic-based semiconductors be merged with imine COFs to fabricate efficient heterostructure photocatalysts for pollutant degradation, but single-site metal can also combine with imine COFs for better photoreactivity. By coordinating metallic Cu into a terpyridine N site in an imine-based COF-909, complete separation of photo-induced electrons and holes can be achieved [42]. The charge carrier renders much improved photodegradation performance of sulfamethoxazole, outperforming the current state-of-the-art catalysts such as P25-TiO_2_ or peroxymonosulfate, with 27 and 40 times higher kinetic constants of COF-909 and P25-TiO_2_ (Figure 6). Supported by the density-function theory (DFT) calculation, the enhanced photoactivity was attributed to the complete separation of excited-state electrons and holes dwelling in the opposite part of the COF-909-Cu backbones in comparison to the overlapped electrons-holes configuration in pristine non-metalated COF-909 materials.

**Figure 7:**
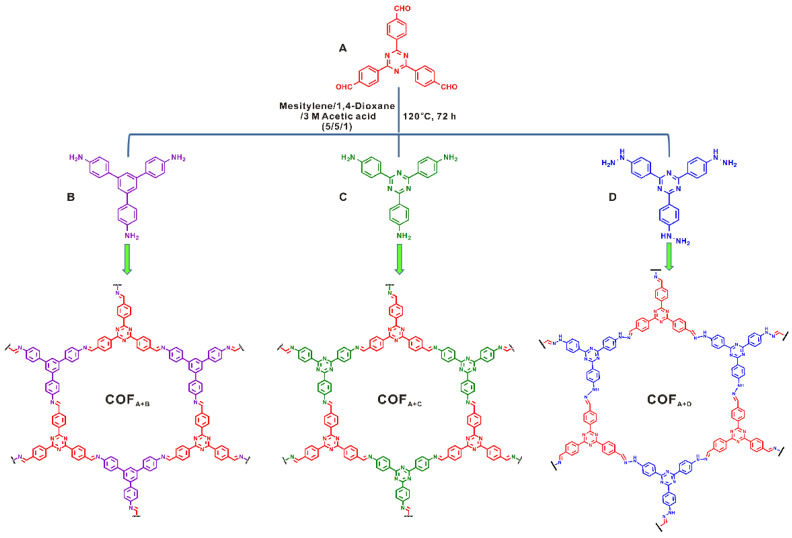
Synthesis of covalent organic frameworks via Schiff base chemistry of different nitrogen-containing building blocks with 4,4′,4″-(1,3,5-triazine-2,4,6-triyl) tribenzaldehyde. (Copied with permission from Elsevier 2018 [43]).

Cai et al. reported that by elaborately screening the monomer electronic and steric structure, a pure-imine-based COF can also provide satisfactory performance for photocatalytic degradation of methyl organic and phenol pollutants in an aqueous solution [43]. They designed and prepared three triazine imine COFs by condensing triazine-based aldehyde with phenylene amine, triazine amine or triazine hydrazine (Figure 7). They discovered that a triazine aldehyde-triazine amine-based imine COF provided the best performance for MO and phenol photodegradation, even though its specific surface area was the least. Supported by the EIS, PL and photocurrent experimental results, the superior performance of the triazine aldehyde-triazine amine COF was attributed to the most photoactive sites and the largest conjugation degree. The triazine ring provided more photoactive nitrogen atoms compared with the phenylene ring, and the triazine-imine COF exhibited much better conjugation in comparison to the triazine-hydrozone COF. Both of these factors leaded to its superior photocatalytic activity.

**Figure 8:**
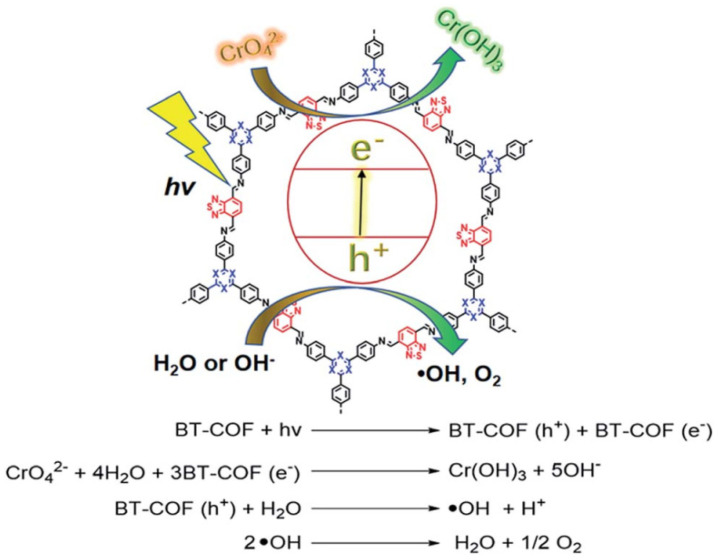
Mechanisms of photocatalytic reduction of Cr (VI) by BT-COF. (Copied with permission from RSC 2019 [44]).

Incorporating an electron donor–acceptor (DA) structure into a COF’s backbone can considerably enhance its light-harvesting and charge transfer ability. Chen et al. reported that benzothiadiazole, which is considered to be the state-of-the-art electron acceptor moiety in photovoltaics, can be included in the BT-COFs photocatalysts [44]. The TPB-BT-COF formed by the Schiff base condensation between *tris*-(4-aminophenyl) benzene (TPB) and benzo[1,2,5]thiadiazole-4,7-dicarbaldehyde (BT)can generate an imine-linked 2D eclipsed AA-stacking structure (Figure 8). The introduction of DA units greatly enhances the visible-light absorbance, widens the absorption spectrum and facilitates the charge generation, separation and migration processes. The as-synthesized TPB-BT-COF exhibited excellent performance upon the photo-reduction of Cr (VI) with the kinetic constant exceeding the current state-of-the-art photocatalyst systems. The boosted activity can be attributed to the effective light harvesting and charge transfer by the electron DA structure.

### 2.3. Ketonenamine-Based COF Photocatalysis for Pollutant Degradation

Although imine-based COFs and their composite COF materials can act as effective and stable photocatalysts for the application of photodegradation of pollutants in aqueous solutions, the imine C=N linkage is still labile and unstable in more acidic or basic conditions, which greatly limits its application in low-or high-pH wastewater treatment. To overcome this issue and further enhance its stability and activity in harsh environments, other robust linkages are designed and developed with various strategies. Among them, ketonenamine-based COFs were the first and the most successful type that can endure strong acidic conditions [45].

**Figure 9:**
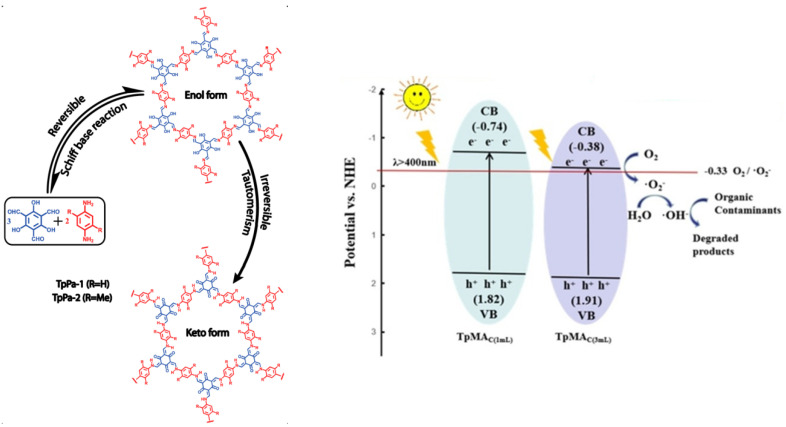
Mechanism of TpMa-COF photodegradation of pollutants. (Copied with permission from ACS 2012 [30] and Elsevier 2019 [46]).

Banerjee et al. initially explored and developed ketonenamine-linked TpPa-COFs in 2012 [30]. They discovered that after the reversible Schiff base condensation between 1,3,5-triformylphloroglucinol and 1,4-phenyldiamine to yield the imine C=N structure, the consequent irreversible enol-ketone transformation generated a stable C=Clinked enamine structure. The first reversible reaction renders the material’s long-order crystallinity, and the second irreversible transformation fulfills the material’s stability to endure acidic conditions. The second irreversible reaction did not destroy the crystallinity since only the bond shifting occurred while the atomic position remained the same (Figure 9 left). Benefitting from the structure ordering and robustness, the TpPa-1 and TpPa-2 COFs exhibited good gas sorption ability and could retain their porosity and crystallinity even after long-term immersing in a strongly acidic 9 M HCl aqueous solution. Furthermore, TpPa-2 COFs can even withstand 9 M NaOH for 7 days without an apparent loss of crystallinity and porosity, since the introduction of sterically hindered methyl groups in aromatic rings prohibited COF’s reactivity towards both nucleophilic and electrophilic attack. The stability of TpPa-1 and TpPa-2towardswater or acid arises from the disappearance of the acid-labile imine (C=N) bond as a result of the irreversible tautomerism, while the stability toward bases arises from the strengthening alkalinity of the secondary nitrogen by two bulky methyl groups that were positioned near the base-labile secondary nitrogen center (C–NH–C=) in TpPa-2 [30]. In a similar way, the hydrophilicity of ketonenamine-based COFs is probably strengthened by the secondary nitrogen of enamine C–NH–C= unit since the polarity of nitrogen in the secondary amine is much larger than that of C=N.

Having benefited from the structural robustness of ketonenamine-based COFs, these types of COFs are extremely suitable for the photocatalytic degradation of aqueous pollutants in a wide pH range. Zhao et al. exploited the mechanochemical approach to prepare TpMa-COF by a ball milling process to facilitate 1,3,5-triformylphloroglucinol and melem with which to proceed with a Schiff base reaction, generating ketonenamine-based COF [46]. The as-prepared COF demonstrated moderate crystallinity and porosity compared to the same COF produced by traditional solvothermal preparation methods. However, its photocatalytic activity is considerably retained for the photodegradation of an aqueous phenol pollutant (Figure 9). Apart from the satisfactory photodegradation performance, the preparation time was greatly reduced. Furthermore, the amount of solvent used in the preparation is decreased by eight-fold. Furthermore, this mechanochemical preparation can be conducted in room temperature and air atmospheres, excluding the harsh and rigorous condition in traditional solvothermal procedures with uncompromised photoactivity.

**Figure 10:**
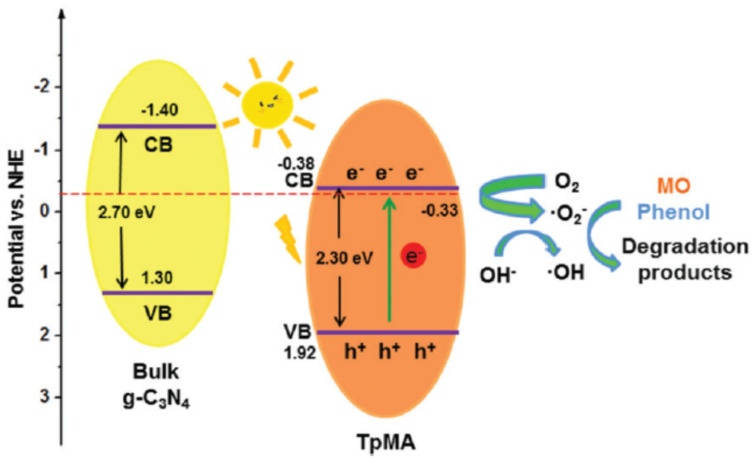
Mechanism of photodegradation of MO and phenol pollutants by TpMa-COF. (Copied with the permission from RSC 2017 [47]).

Cai et al. designed and prepared TpMa-COF by condensing 1,3,5-triformylphloroglucinol and melamine via a reversible Schiff base reaction and the following irreversible enol-ketone tautomerization [47]. The as-synthesized TpMa-COF possessed a C_3_N_4_-unit, which rendered the COF photocatalytic activity (Figure 10). Compared with the pristine bulk C_3_N_4_ material, the TpMa-COF displayed a narrower band gap, enhanced light absorption, a wider absorption range, boosted electron–hole generation, transfer and separation activity. Furthermore, this COF photocatalyst exhibited more satisfactory performance for the photodegradation of methyl orange (MO) and phenol pollutants compared with bulk C_3_N_4_. Moreover, due to the irreversible formation of the ketonenamine structure, the TpMa-COF exhibited dramatically enhanced stability in water, basic and even acidic conditions. The as-prepared TpMa-COF can maintain its photocatalytic activity without apparent loss even after five cycles. This is the first report of a ketonenamine-based COF for photodegradation applications.

Besides pure-ketonenamine-based robust COF photocatalysts, composite photocatalyst systems combining ketonenamine and other inorganic or organic photoactive compounds can be a promising strategy for better photodegradation performance. The structural robustness of ketonenamine COFs offers better opportunity to hybridize with other semiconductor photocatalysts via pre- and post-modification. Accordingly, Zhang et al. discovered that the ketonenamine-based Tp-Ta-COF can act as very effective substrate to grow Fe-doped ultra-small 2.3 nm TiO_2_ nanoparticles on it [48]. The use of ketonenamine as a photocatalyst support exhibited multiple advantages, such as its capability to assist the controlled growth of ultra-small-sized photocatalyst nanoparticles, the better adsorption of organic pollutants on its numerous inner sites, the facilitated mass transfer process for pollutant transmission into the materials and the innocuous product desorption into the bulk solution. The as-prepared 5-Fe-TiO_2_@COF displayed the best performance towards photodegradation of methylene blue (MB) dye pollutant under ambient solar light irradiation, exceeding the pristine Fe-TiO_2_ by eight-fold, while the pristine Tp-Ta-COF did not show any photoactivity towards MB degradation. 

**Figure 11:**
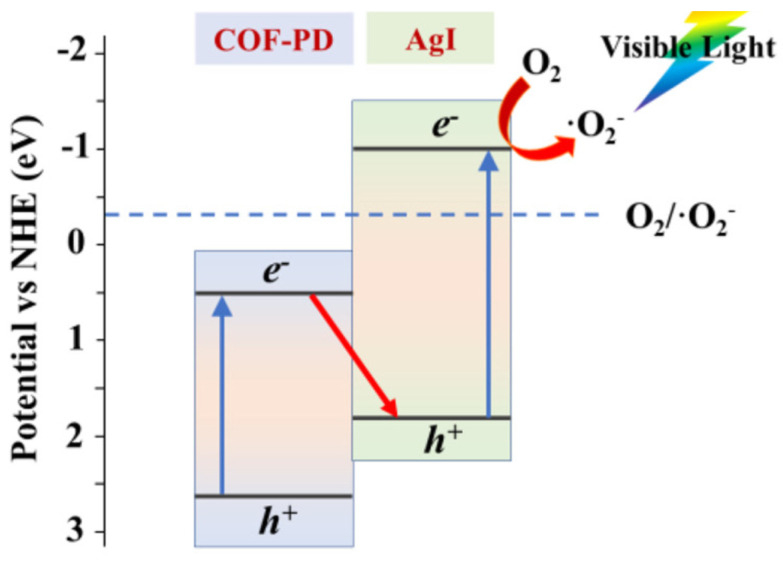
Mechanism of the formation of Z-scheme COF-PD and AgI photocatalyst (Copied with permission from Elsevier 2020 [49]).

Tong et al. discovered that ketonenamine-based COF-PD can combine with AgI to form an effective visible-light-driven photocatalyst that can kill and degrade bacteria and organic pollutants in an aqueous solution [49]. The AgI nanoparticles were grown in the outer shell of COF-PD. The COF-PD@AgI hybrid photocatalyst was demonstrated to possess the ability to kill *Escherichia coli* bacteria and degrade RhB and acetaminophen organic pollutants. Supported by the scavenger quenching experiments and electron paramagnetic resonance results, the main active species for *E. coli* disinfection, RhB and acetaminophen degradation are different. During the photocatalytic degradation, O_2_^−^^•^ plays the pivotal role while ^•^OH contributes greatly to the *E. coli* disinfection, but little to the degradation of organics. The reason for this is that the small amounts of ^•^OH can attack the bacterial cell membrane and lead to its death, but there is not enough for the considerable degradation of RhB and acetaminophen. Moreover, from the VB XPS and DRS band gap measurements, the authors determined that the COF-PD@AgI did not form the traditional type II heterojunction since the CB potential of COF-PD cannot reduce O_2_ molecules but yield a dual-band Z-scheme heterojunction (Figure 11). The CB electrons on AgI can reduce O_2_ to generate O_2_^−^ and secondary ROS, while the VB holes on AgI can combine with CB electrons on COF-PD. Thus, the VB holes on COF-PD can be more active because of the efficient trap of its CB electrons by AgI. This would enhance its oxidative ability to react with pollutants, substrates or H_2_O molecules. This dual-band Z-scheme heterojunction strategy is considerably effective for pollutant photodegradation applications.

**Figure 12:**
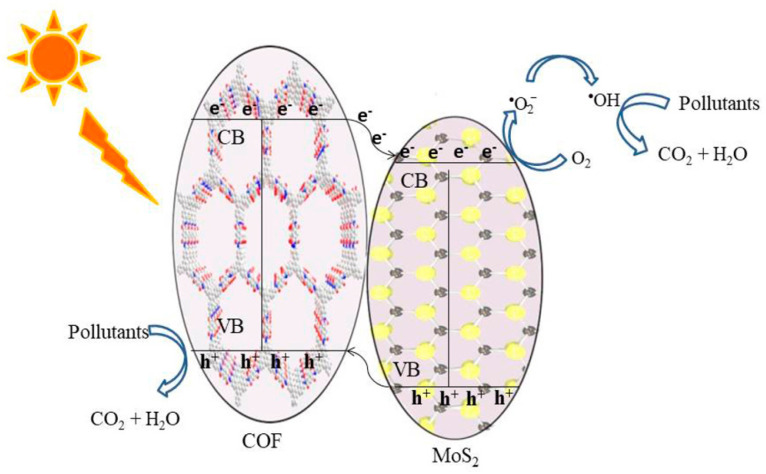
Schematic illustration of the photocatalytic degradation by the TpPa-1 COF/MoS_2_ heterojunction photocatalyst. (Copied with permission from ACS 2020 [50]).

Apart from metal-oxide and halide semiconductor photocatalysts, recently, transition metal-disulfides (TMD)-based materials become the focus in materials chemistry and catalysis due to its excellent charge transfer property and electron mobility, as well as its ability as an effective hydrogen-evolving reaction (HER) catalyst. Zhang et al. exhibited that narrow-band semiconductor MoS_2_ can hybridize with a ketonenamine TpPa-1 COF to effectively photodegrade tetracycline and RhB pollutants (Figure 12) [50]. The 2D MoS_2_ component greatly promoted charge separation and mobility and prohibited the charge recombination by the formation of a heterojunction with TpPa-1 COF. This is the first example that combines non-noble metal 2D materials with COFs for efficient photodegradation applications.

**Figure 13:**
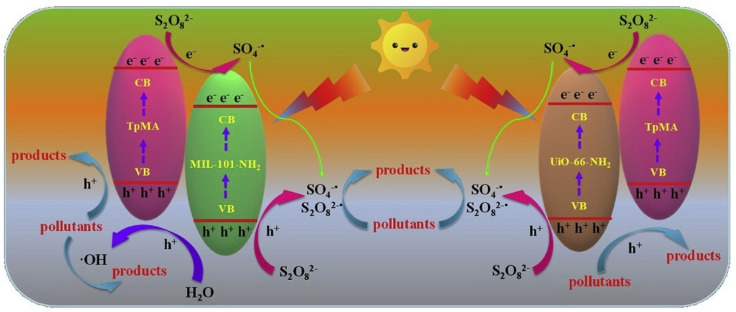
Mechanism of the photodegradation by the MOF@COF/PS composite photocatalyst system. (Copyright with permission from Elsevier 2020 [51]).

Besides common inorganic semiconductor combination with ketonenamine-based stable COF photocatalysts, inorganic-organic hybrid MOFs can also hybridize with ketonenamine COFs for photodegradation applications. Wang et al. reported that Fe-based MIL-101-NH_2_ and Zr-based UiO-66-NH_2_ MOFs can incorporate with ketonenamine TpMa-COF to yield two novel MOF@COF photocatalysts [51]. The as-prepared hybrid photocatalysts possessed enhanced visible-light absorption and better photo-generated electron-hole separation properties. These hybrid materials acted as highly effective photocatalysts for the activation of persulfate (PS) for the degradation of the bisphenol A (BPA) endocrine disruptor (Figure 13). The composite MOF@COF/PS photocatalyst systems exhibited much boosted performance in comparison to the pristine MIL-101-NH_2_, UiO-66-NH_2_ and TpMa-COF. Supported by the radical trapping experiments, h^+^ and ^•^OH played the pivotal roles, while O_2_^−^^•^ did not participate in the degradation processes.

Apart from inorganic and inorganic-organic hybrid semiconductor photoactive materials, pure organic semiconductor g-C_3_N_4_ can also combine with stable ketonenamine-based COFs for photo-detoxification applications in an aqueous solution. Jin et al. designed and synthesized a ketonenamine-based triazine COF by condensing Tp (1,3,5-triformylphloroglucinol) with 1,3,5-*tris* (4-aminophenyl)-triazine [52]. Furthermore, the triazine COF can hybridize with g-C_3_N_4_ by co-condensation of the COF precursors with melem. The triazine-COF-g-C_3_N_4_ hybrid materials exhibited enhanced the visible-light absorption range to 850 nm and much increased the specific surface area from 58 to 558 m^2^/g. The increased specific surface area means enhanced adsorption capability for organic pollutants. The photodegradation rate for RhB under visible-light irradiation by the hybrid photocatalyst is 15 and 1.8 times as compared to the pristine triazine COF and bulk g-C_3_N_4_.

**Figure 14:**
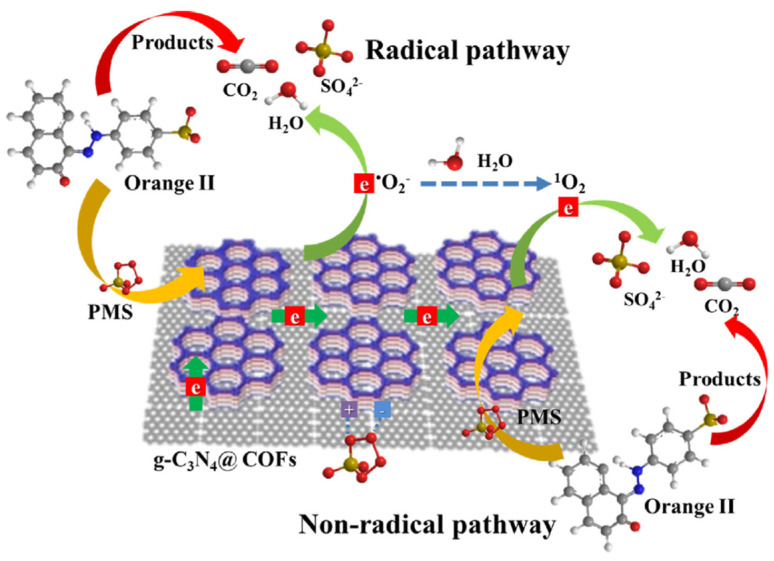
Schematic illustration of radical and non-radical pathway of photodegradation of Orange II dyes by g-C_3_N_4_@COFs/PMS photocatalyst. (Copied with permission from Elsevier 2019 [53]).

Furthermore, Wang et al. reported that ketonenamine-based COF can also combine with g-C_3_N_4_ to activate peroxymonosulfate for effective photodegradation of organic dyes pollutants [53]. Ketonenamine-based TpPa-1-COF was fabricated by condensing Tp with 1,4-phenyldiamine. The as-synthesized TpPa-1-COF was composited with g-C_3_N_4_ by a simple immersing and annealing process. The hybrid materials demonstrated enhanced specific surface area, nitrogen content in addition to mass and charge transfer. The UCN@COF photocatalyst fabricated from TpPa-1-COF with urea-derived carbon nitride exhibited most satisfactory activity towards photodegradation of refractory Orange II dye pollutants in aqueous solution. Interestingly, the active species for dye degradation of this photocatalyst system is greatly varied from other common COF- and g-C_3_N_4_-based photocatalysts. Supported by the radical scavenger quenching and EPR trapping experiments, a singlet O_2_ was discovered to be the main contributing species for degradation, while other commonly met ROS included ^•^OH and O_2_^−^^•^. The ^1^O_2_ and persulfate radical substantially attack and destruct the organic dye pollutants (Figure 14). 

### 2.4. Miscellaneous COFs Photocatalysis for Pollutant Degradation

Besides common imine- and ketonenamine-based COF photocatalysts, COFs with other stable linkages were also developed for photodegradation applications. To name a few, conjugated sp^2^-C=Clinked olefin COFs [54] and phenyl-triazine C–C linked covalent-triazine-frameworks (CTFs) [55] exhibited promising potential for the photodegradation of aqueous pollutants because of their high conjugation degree and extreme stability towards aqueous solution. The overall conjugation renders much convenient photo-generated charge transfer channels and increased electron mobility. The C=C and C–C linkages are stable since they are less polar than their imine analogues. Thus, COFs and CTFs with these two linkages are appropriate for photocatalytic applications in an aqueous solution.

**Figure 15:**
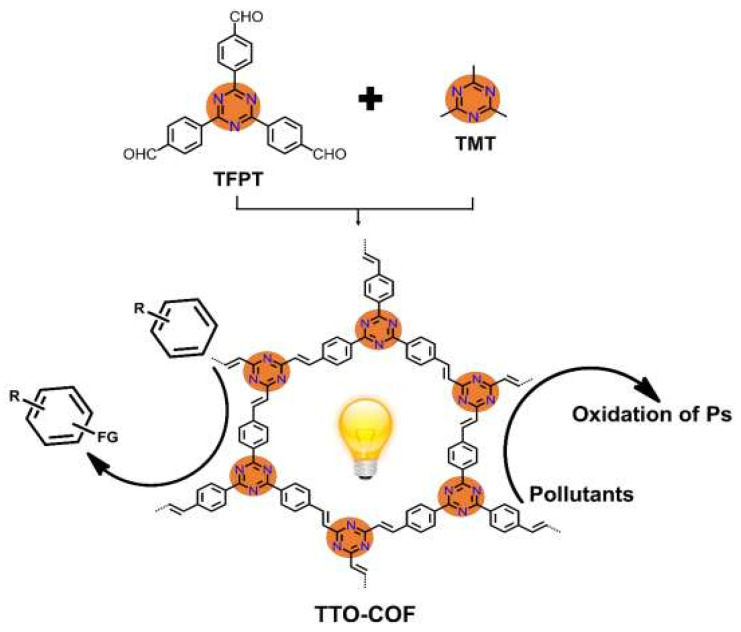
The preparation and photocatalytic applications of sp^2^-C conjugated TTO-COF. (Copied with permission from Elsevier 2020 [56]).

Cai et al. reported that by a reversible aldol reaction, 2,4,6-tris(4-formyl-phenyl) -1,3,5-triazine (TFPT) and 2,4,6-trimethyl-1,3,5-triazine (TMT) can form a triazine-based sp^2^-C=C linked olefin COF [56]. The as-synthesized olefin COF exhibited ultra-high stability in extreme basic and acidic conditions (Figure 15). Moreover, the fully conjugated olefin COF showed much enhanced electron-hole transfer kinetics compared with their imine COF and g-C_3_N_4_ analogues. This COF can absorb the visible light to initiate the photodegradation of methyl orange and methylene blue dye pollutants with high efficiencies. As indicated by EPR trapping experiments and radical scavenger quenching results, O_2_^−^^•^ played a pivotal role in degradation process, while ^•^OH and h^+^ did not make considerable contributions. Moreover, this olefin COF can be directly exploited to photocatalyze the C–H trifluomethylation of arenes and heteroarenes with CF_3_SO_2_Na (Langlois reagent) by direct activation of COF without the participation of commonly met ROS. This robust olefin COF displayed the capability for C=C linked fully conjugated COFs for photodegradation and photocatalytic organic synthesis potential.

**Figure 16:**
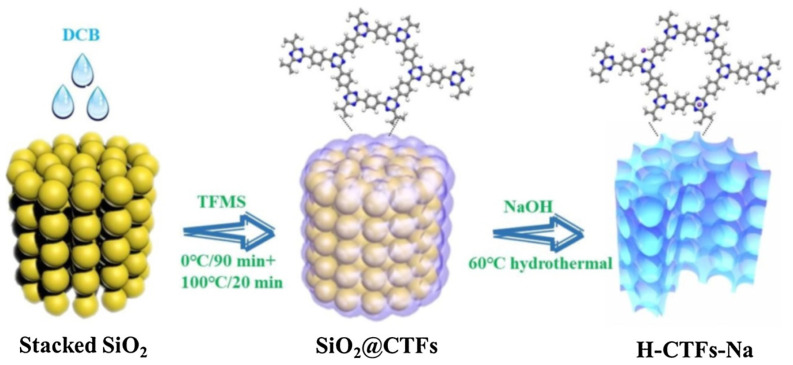
Schematic illustration of template-assisted preparation of H-CTF-Na photocatalyst. (Copied with permission from Elsevier 2019 [57]).

As nitrogen-rich framework-based materials, covalent triazine frameworks (CTFs) have shown their promising applications for photocatalysis, including water-splitting, CO_2_ reduction and organic synthesis. Owing to its stable and robust C–C linkages, CTFs are also well fitted for aqueous photocatalytic degradation applications. Their abundant nitrogen sites can help to enhance the photo-harvesting and charge transfer properties, while the C–C linkage grants them ultra-high stability in basic, acidic and strongly irradiated conditions. Song et al. reported that CTF-1 synthesized by a low-temperature CF_3_SO_3_H catalytic procedure can be further shaped into a 3D honeycomb nanostructure by incorporating a SiO_2_ template [57]. Furthermore, sodium was doped through NaOH etching the SiO_2_ template (Figure 16). The as-prepared H-CTF-Na exhibited enhanced light harvesting and a narrower band gap because of considerable Na doping. This H-CTF-Na photocatalyst can activate peroxymonosulfate under sunlight irradiation to photodegrade refractory carbamazepine (CBZ) pollutants. The H-CTF-Na/Vis/PS system displayed much boosted performance for CBZ degradation than the H-CTF-Na/Vis and H-CTF-Na/PS system. From various radical scavenging quenching and comparative EPR trapping studies, the authors discovered that h^+^, ^•^OH and SO_4_^•^^−^ are the main reactive species responsible for CBZ degradation. Moreover, the photocatalyst system also showed versatility towards multiple phenols and organic dye pollutants and considerable stability for five consecutive runs without apparent loss of activity. The artful design of the 3D honeycomb hollow nanostructure strongly increased the light-harvesting and utilization properties. Furthermore, collaboration with the PS activation enhanced the separation and transfer of photo-generated h^+^ species and lead to the more convenient generation of ^•^OH and SO_4_^•^^−^ reactive species for pollutant degradation.

All of the COF photocatalysts reported for degradation of pollutants in water are summarized in Table 1, including their efficiency, band gap and specific surface area.

## 3. Conclusions, Perspectives and Opportunities

Although still in its infancy stage, COFs and COF-based composite materials have demonstrated their potential in the field of aqueous pollutant degradation under visible-light or ambient sunlight irradiation. They have garnered considerable successes in this area, but there still remain some concrete challenges in COF photocatalytic materials designing and synthesizing required to be overcome. Firstly, the general method to grow large size 2D COF single crystals appropriate for the common single-crystal X-ray diffractometer measurements is still essentially desired [58]. Since the main COFs applied in the photocatalytic degradation are 2D COFs, the direct establishment of the structure-activity relationship is of great significance. The most effective way to obtain the structure-activity relationship is to utilize the single-crystal X-ray diffraction to unambiguously assign the intralayer chemical composition and the interlayer stacking mode. However, owing to the great difficulty to realize the controllable ordered growth with simultaneously tuning of covalent bonds in the intralayer *a*- and *b*-axis direction and the van der Waals interaction in the interlayer *c*-axis direction, 2D COF crystallization issues still remain a great obstacle for the clarification of the structure-activity relationship, albeit 3D single-crystal COFs can be fabricated by the addition of a mono-functional aniline modulator [59]. Secondly, the light-harvesting range of COF-based photocatalysts needs to be further broadened. In particular, the near-infrared (NIR) light absorption should be strengthened. Since NIR light accounts for almost half of the total sunlight energy, the effective utilization of NIR light is the key to increase the solar energy exploitation efficiency. However, the absorption edge of most current COF photocatalysts is centered at the visible-light region, without apparent absorption above 700 nm [60]. Moreover, even if part of NIR light can be absorbed, the low-energy NIR photon usually could not trigger the cleavage of common C-H, C-C and C-O chemical bonds, leading to no photoactivity. To overcome this issue, electron donor–acceptor (DA) structure should be incorporated into the COFs backbones to produce a long-wavelength charge transfer absorption band [61]. Additionally, the heterojunction structure can be built by merging with other photoactive materials such as metal-oxides, metal sulfides, organometallic compounds, MOFs and carbon nitrides [38]. The formation of type II or Z-scheme heterojunctions can widen the light-harvesting spectrum. Two-proton-absorption (TPA) monomers should be included in the COF skeletons to effectively transform the low-energy NIR photon into a reactive high-energy visible-light photon to initiate the chemical bond cleavage [62]. Thirdly, the degradation efficiency should be enhanced for the practical application of the realistic large-scale water treatment project. More effective and reusable membrane-based COF photocatalyst materials should be used other than COF powder photocatalysts. Furthermore flow-bed reactors should substitute the batch reactors to increase the spatial-time degradation yield. Owing to the non-solubility in nearly all organic solvents, the solution-based process of transforming COF powders into uniform COF membranes is challenging [63]. Finally, accurate recognition and selective photodegradation of targeted pollutants from actual water is one of the best approaches for COF materials to boost degradation efficiency, albeit it is very challenging. The shape-selective channels and anchoring sites of COFs can be designed to fit targeted pollutant structures and sizes very well. This favors targeted pollutant molecules over solvents and other substrates to access the reactive sites and react with photo-induced h^+^ of COFs, thereby promoting their practical feasibility in the degradation of trace but highly toxic pollutants in water.

Although there still remains many challenges and issues, we do think the future of COF materials for aqueous pollutants photodegradation will be fruitful only if the related chemistry community continues to devote their efforts on this burgeoning area.

## Figures and Tables

**Table 1:** COF-based photocatalysts for photocatalytic degradation of different aqueous pollutants.

COF	Specific Surface Area (m^2^/g)	Band Gap (eV)	Pollutant	Degradation Rate Constant (min^−1^)	Ref.
NH_2_-MIL-68@TPA-COF	539	2.21	RhB	0.077	[38]
Fe_3_O_4_@MOF_UiO-66_@Tz-Dz-COF	1279	3.68	Mg/CR	1.21/0.65	[39]
La^3+^, Sb^3+^-doped MOF-In_2_S_3_@FcDc-TAPT COFs	NA	NA	RhB/Cr (VI)	0.01935/0.05876 (La) 0.03556/0.4307 (Sb)	[40]
CuPor-Ph-COF@g-C_3_N_4_	NA	2.56	RhB	0.021	[41]
COF-909 (Cu)	1959	1.0	SMX	0.133	[42]
COF_A+C_	1903	2.56	MO/phenol	0.09 (MO)	[43]
TPB-BT-COF	1376	2.05	Cr (VI)	99%	[44]
TpMa-COF	NA	2.29	MO	0.044	[46]
TpMa-COF	56.9	2.30	MO/phenol	0.102 (MO)	[47]
Z-Scheme COF-PD/AgI	373.3	2.18/2.80	*E. coli*/RhB/ACTP	0.0466 (RhB)/ 0.0204 (ACTP)	[49]
TpPa-1 COF/MoS_2_	18.984	1.59	RhB/TC	0.118 (RhB)/ 0.0316 (TC)	[50]
MOF@COF/PS	129.2	2.12	BPA	0.011	[51]
g-C_3_N_4_@COFs/PMS	96.4	NA	Dyes	0.10 (Orange II)	[53]
TTO-COF	390.35	2.46	MO/MB	0.1938 (MB)/ 0.159 (MO)	[56]
H-CTF-Na	18.6	2.88	CBZ	0.0302	[57]

## Data Availability

Not applicable.
